# Fear of falling and all-cause mortality among young-old community-dwelling adults: a 6-year prospective study

**DOI:** 10.1007/s10433-021-00635-5

**Published:** 2021-07-03

**Authors:** Giulia Belloni, Christophe Büla, Brigitte Santos-Eggimann, Yves Henchoz, Sarah Fustinoni, Laurence Seematter-Bagnoud

**Affiliations:** 1 Service of Geriatric Medicine and Geriatric Rehabilitation, University of Lausanne Hospital Centre, Mont-Paisible 16, CH-1011 Lausanne, Switzerland; 2 Health Services Unit, Center for Primary Care and Public Health (Unisanté), University of Lausanne, Lausanne, Switzerland

**Keywords:** Falling concern, FES-I, Older adults, Death

## Abstract

This study investigated whether fear of falling (FOF) measured by two different instruments, the Falls Efficacy Scale-International (FES-I) and the single question on FOF and activity restriction (SQ-FAR), is associated with mortality at 6-year follow-up. Participants (*n* = 1359, 58.6% women) were community-dwelling persons enrolled in the Lausanne cohort 65 + , aged 66 to 71 years at baseline. Covariables assessed at baseline included demographic, cognitive, affective, functional and health status, while date of death was obtained from the office in charge for population registration. Unadjusted Kaplan Meyer curves were performed to show the survival probability for all-cause mortality according to the degree of FOF reported with FES-I and SQ-FAR, respectively. Bivariable and multivariable Cox regression analyses were performed to assess hazard ratios, using time-in-study as the time scale variable and adjusting for variables significantly associated in bivariable analyses. During the 6-year follow-up, 102 (7.5%) participants died. Reporting the highest level of fear at FES-I (crude HR 3.86, 95% CI 2.37–6.29, *P* < .001) or “FOF with activity restriction” with SQ-FAR (crude HR 2.42, 95% CI 1.44-4.09, *P* = .001) were both associated with increased hazard of death but these associations did not remain significant once adjusting for gender, cognitive, affective and functional status. As a conclusion, although high FOF and related activity restriction, assessed with FES-I and SQ-FAR, identifies young-old community-dwelling people at increased risk of 6-year mortality, this association disappears when adjusting for potential confounders. As a marker of negative health outcomes, FOF should be screened for in order to provide personalized care and reduce subsequent risks.

## Introduction

Fear of falling (FOF) is common in community-dwelling older people, and has been shown to be associated with adverse outcomes such as frailty incidence, care dependence, depression, or falls (Scheffer et al. 2008; Seematter-Bagnoud et al. 2010). Death might be another adverse event linked to fear of falling, and any evidence of an independent association between FOF and middle-term mortality would further justify more efforts in screening for and intervening on fear of falling.

To our knowledge only few studies evaluated FOF as a marker of increased risk for mortality, and all were carried out in an Asian population. Three of them found a significant independent association between FOF, assessed with a single question, and mortality among community-dwelling people during a follow up ranging from 7 to 10 years (Chang et al. 2017; Kim & Bae 2020; Lee et al. 2020). This association was observed when adjusting for socio-demographic variables, lifestyle habits as well as chronic diseases. On the other hand, Oh et al.’s study found that the association with mortality was not significant anymore when adjusting multivariable models for chronic diseases in addition to socio-demographic and lifestyle habits (Oh et al 2019). A stronger relationship was observed in men than in women in the two studies that provided sex-specific results (Chang et al. 2017; Lee et al. 2020). Although two studies were able to examine the association of low, respectively moderate FOF with mortality, showing a dose-response gradient, none of them measured FOF-related activity restriction. This precluded to further evaluate whether FOF with activity restriction would be more strongly associated with mortality than FOF alone, through a pathway of physical inactivity, increased sarcopenia, and frailty over time (Choi et al. 2017; Deshpande et al. 2008). Further insight on such an association would help better targeting of older persons needing intervention on fear of falling, as well as better designing of such interventions.

The aim of this study was to prospectively examine the relationship between self-reported FOF at baseline, and all-cause mortality at 6-year follow-up, among young-old community-dwelling persons. Two instruments were used to assess FOF, the 16-item FES-I that allows an in-depth assessment of activity-related FOF, as well as the SQ-FAR, a quickly administered tool that includes activity restriction assessment (Belloni et al. 2020b). We hypothesized that highest levels of FOF and FOF with activity restriction, would be associated with increased risk of mortality.

## Methods

### Study design and population

Data were obtained from the Lausanne cohort 65 + (Lc65 +), whose design has been described in detail elsewhere (Santos-Eggimann et al. 2008). Briefly, a population-based sample of 1564 residents of Lausanne aged 65–70 years were enrolled in the cohort in 2004 from the 3056 persons who were contacted (51.2%). Distribution of birth year, sex, as well as of some socio-economic characteristics were similar in participants as in aggregate statistics from the national population census for the target population. In 2005, 1524 persons were still eligible and 1422 (90.9%) answered the yearly postal questionnaire. Follow-up also includes an in-person visit with physical examination and performance tests every three years from 2005 on.

The present study includes data from 1359 community-dwelling participants born between 1934 and 1938 who completed the 2005 baseline assessment and who answered the SQ-FAR as well as the FES-I.

The Lc65 + study received approval from the Cantonal Human Research Ethical Committee (Protocol 19/04). Participants were informed of the study goals and design, and gave written consent.

### Mortality

All-cause mortality data, from the initial assessment in 2005 through 31st December 2011, were obtained from the office in charge for population registration in the Canton of Vaud.

### Exposure variable: fear of falling assessment at baseline

Two FOF measures were included in the self-completed questionnaire:

- The FES-I is based on self-reported concern about falling while performing 16 activities of daily living (such as going up and down stairs, or getting dressed or undressed). Four response options are possible, ranging from “not at all concerned” to “very concerned”, with a total score ranging from 16 to 64. Persons who did not answer to all items were excluded. Following Delbaere et al. cut-points, three levels of FOF were considered: low (score 16–19), moderate (score 20–27), and high (score 28–64) (*FES-I*, n.d.; Delbaere et al. 2010).
- The SQ-FAR was a combination of the answers to the question “Are you afraid of falling?“ and “If yes, have you restricted any activities because of this fear?”(Belloni et al. 2020b). Three levels of FOF were considered: not afraid (no FOF), afraid without activity restriction (FOF without AR), and afraid with activity restriction (FOF with AR). This tool has demonstrated a moderate agreement (87.8%, Kappa 0.57) with FES-I in the same population (Belloni et al. 2020b).

### Covariates

Information on the following variables was collected by the 2004 and 2005 self-completed postal questionnaires: multimorbidity (defined as 2 or more chronic diseases out of a list of 12), higher education (defined as 12 or more years of education), visual impairment (defined as difficulty reading a newspaper), number of falls in the last 12 months (none, one, two or more), difficulty or help needed in basic activities of daily living (BADL: dressing, bathing, eating, getting in /out of the bed/armchair and using the toilet), as well as in instrumental activities of daily living (IADL: doing usual household chores or going shopping) and depressive symptoms (defined as at least one positive answer to two screening questions on anhedonia and sadness in the last 4 weeks) (Whooley 2016).

Data collected during the 2005 in-person visit included cognitive assessment using Folstein’s Mini Mental State Examination (MMSE, a score < 24/30 defining cognitive impairment, (Folstein et al. 1975) and frailty assessment. Frailty was assessed according to Fried’s frailty phenotype (Fried et al. 2001) (prefrail = 1 or 2 criteria; frail ≥ 3 criteria), with minimal adaptation of three criteria (shrinking, poor endurance, low activity) (Santos-Eggimann et al. 2008).

### Analyses

Baseline population characteristics were reported according to the occurrence of death during the follow-up.

Unadjusted Kaplan Meyer curves were performed to show the survival probability for all-cause mortality according to the degree of FOF reported with FES-I and SQ-FAR, and were compared using the log rank test.

Bivariable cox regression analyses were employed to assess unadjusted hazard ratios, using time-in-study as the time scale variable and adjustment for age. Due to a few missing data, sample size was 1256 (92.3%) for analyses using variables collected during interview (frailty and cognitive impairment) and higher than 1300 (> 95%) for all other variables.

In order to evaluate whether FOF was independently associated with all-cause mortality at a 6-year follow-up, two multivariable cox regression models, one for each independent variable of interest (FES-I or SQ-FAR), were performed. Covariates were selected based on their known association with fear of falling and mortality risk such as reported on the literature, and adjustment variables were selected based on their significant association with mortality in bivariable analyses. Given the narrow age range of the participants, the association with age was not significant, and models were not age-adjusted. The absence of collinearity between exposure and adjustment variables was tested using the variance inflation factor (VIF) (Chatterjee et al. 2000).

The assumptions of proportional hazards were verified using log–log plots as well as Schoenfeld residuals. As supplementary material, the same analyses were performed after stratification for sex. All statistical analyses were conducted using Stata, version 14.0.

## Results

Baseline characteristics of participants according to the occurrence of death are shown in Table 1. Among 1359 participants, 102 (7.5%) died during the 6-year period.

**Table 1:** Baseline characteristics of the study population according to the occurrence of death and results of bivariable Cox regression

Characteristics	Death during the 6-year follow-up?		Unadjusted hazard	
	No (*N* = 1257)	Yes (*N* = 102)	Ratio (95% CI)	*P* value^*^
Age (years; mean ± SD)	69.0 ± 1.4	68.9 ± 1.4	0.94 (0.82–1.09)	.423
Women *n* (%)	749 (59.6)	47 (46.1)	**0.60 (0.41–0.88)**	**.001**
Higher education *n* (%)	439 (34.9)	32 (31.4)	0.87 (0.57–1.32)	.515
Comorbidity (2 + chronic diseases) *n* (%)	533 (42.4)	47 (46.1)	1.20 (0.81–1.78)	.354
Depressive symptoms *n* (%)	300 (23.9)	28 (27.5)	1.22 (0.79–1.89)	.368
Cognitive impairment *n* (%)	58 (4.6)	9 (8.8)	**2.16 (1.08–4.31)**	**.029**
Visual impairment *n* (%)	179 (14.2)	24 (23.5)	**1.85 (1.17–2.93)**	**.009**
Falls in the last 12 months *n* (%)				
None	1037 (82.5)	84 (82.4)	Reference	
One	157 (12.5)	10 (9.8)	0.80 (0.41–1.54)	.497
Two or more	63 (5.0)	8 (7.8)	1.56 (0.76–3.23)	.227
BADL impairment *n* (%)	121 (9.6)	19 (18.6)	**2.12 (1.29–3.49)**	**.003**
IADL impairment *n* (%)	176 (14.0)	31 (30.4)	**2.61 (1.71–3.98)**	** < .001**
Frailty and pre-frailty *n* (%)	315 (25.1)	43 (42.2)	**2.57 (1.69–3.91)**	** < .001**
FES-I				
Low concern of falling	887 (70.6)	61 (59.8)	Reference	
Moderate concern of falling	295 (23.5)	19 (18.6)	0.94 (0.56–1.58)	.821
High concern of falling	75 (6.0)	22 (21.6)	**3.86 (2.37–6.29)**	** < .001**
SQ-FAR *n* (%)				
No FOF	855 (68.0)	63 (61.8)	Reference	
FOF without AR	304 (24.2)	21 (20.6)	0.94 (0.57–1.54)	.801
FOF with AR	98 (7.8)	18 (17.7)	**2.42 (1.44–4.09)**	**.001**

Results in bold are statistically significant (*P*-value < .05)

Note: Sample size was 1256 for frailty and cognitive impairment data, and higher than 1300 for all other variables

^*^Bivariable Cox regression

**Fig. 1:**
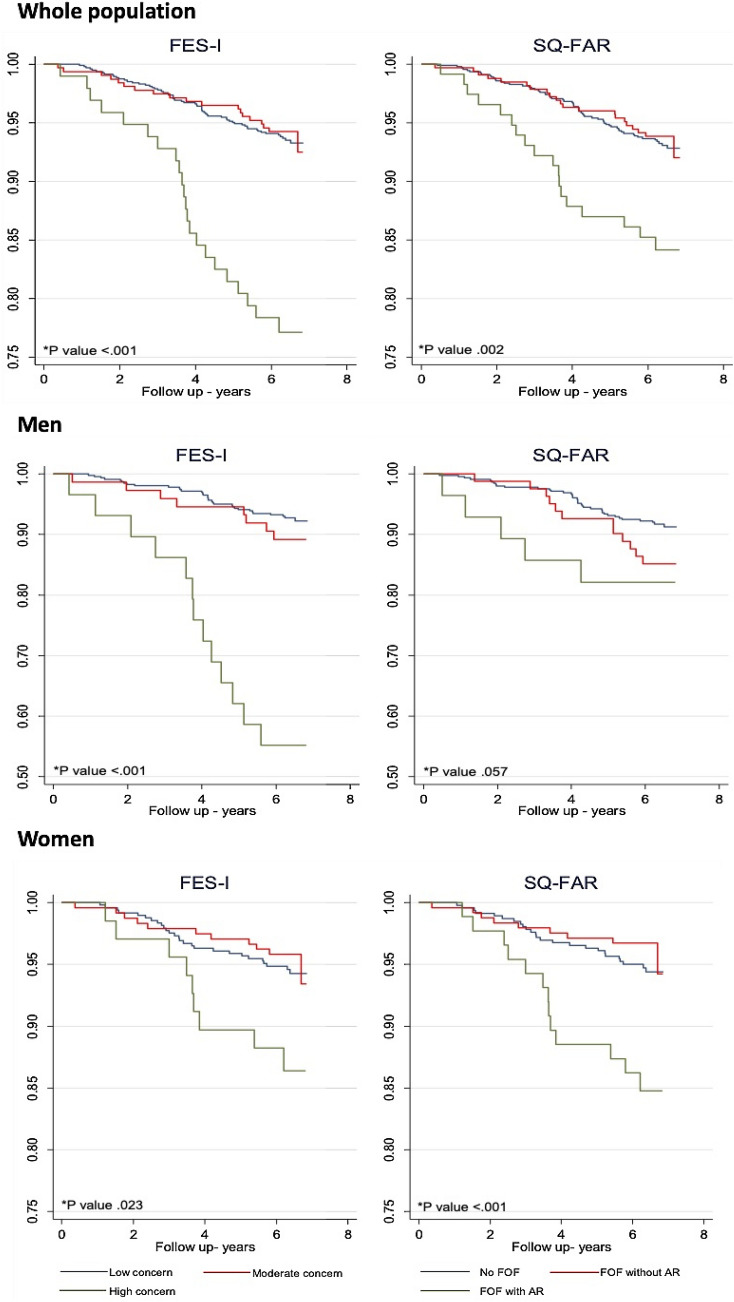
Kaplan-Meyer survival curves for mortality *P value from log rank test

In bivariable cox regression analyses, reporting higher levels of FOF with FES-I (Crude HR 3.86, 95% CI 2.37–6.29, *P* < 0.001) and FOF with AR using SQ-FAR (Crude HR 2.42, 95% CI 1.44–4.09, *P* = 0.001) were both associated with increased risk of 6-year mortality (Table 1). Figure 1 shows Kaplan-Meyer survival curves for mortality based on baseline FOF level among all participants and according to gender. Covariates significantly associated with mortality in bivariable analyses using log rank test were gender, cognitive impairment, visual impairment, BADL and IADL impairment, and frailty (Table 1). There was no collinearity between FOF as measured by FES-I, respectively SQ-FAR, and covariates, with VIF values smaller than 2. When adjusting for this set of variables, neither FES-I nor SQ-FAR did remain significantly associated with mortality at 6-year in multivariable analyses, whereas being a woman, reporting IADL impairment and being frail were associated with a higher mortality risk in both FOF models (Table 2).

**Table 2:** Multivariable cox regression of the association between fear of falling at baseline and mortality during 6-year follow up

	FES-I		SQ-FAR	
	Hazard ratio (95% CI)	*P* value	Hazard ratio (95% CI)	*P* value
FES-I			–	
Low concern of falling	Reference			
Moderate concern of falling	0.78 (0.42–1.44)	.424		
High concern of falling	1.98 (0.91–4.33)	.087		
SQ-FAR	**–**			
No FOF			Reference	
FOF without AR			0.89 (0.50–1.58)	.685
FOF with AR			1.49 (0.73–3.07)	.276
Women	**0.47 (0.30–0.74)**	**.001**	**0.45 (0.28–0.71)**	**.001**
Cognitive impairment	1.46 (0.68–3.15)	.333	1.62 (0.76–3.41)	.209
Visual impairment	1.23 (0.72–2.12)	.454	1.24 (0.73–2.13)	.429
BADL impairment	0.73 (0.35–1.50)	.389	0.82 (0.40–1.66)	.579
IADL impairment	**2.15 (1.08–4.28)**	**.030**	**2.25 (1.14–4.40)**	**.019**
Frailty and pre-frailty	**1.96 (1.20–3.21)**	**.007**	**1.95 (1.20–3.18)**	**.007**

Results in bold are statistically significant (*P*-value < .05)

Note: Sample size was 1230, with 83 deaths

Sex-specific analyses were performed even though the interaction between FOF measures and sex was not statistically significant. A highest level of FOF at FES-I (Adj HR 3.89, 95% CI 1.27-11.94, *P* = 0.017) increased the hazard of death only among men (Tables 3 and 4 in the “appendix”).

**Table 3:** Multivariable cox regression of the association between fear of falling at baseline and mortality during 6-year follow-up in men

	FES-I		SQ-FAR	
	Hazard ratio (95% CI)	*P* value	Hazard ratio (95% CI)	*P* value
FES-I			–	
Low concern of falling	Reference			
Moderate concern of falling	1.25 (0.56–2.79)	.593		
High concern of falling	**3.89 (1.27- 11.94)**	**.017**		
SQ-FAR	–			
No FOF			Reference	
FOF without AR			1.15 (0.54–2.45)	.710
FOF with AR			1.00 (0.29- 3.39)	.997
Cognitive impairment	1.51 (0.55–4.13)	.421	2.43 (0.99–5.95)	.052
BADL impairment	0.43 (0.14–1.31)	.138	0.63 (0.21–1.85)	.398
IADL impairment	1.83 (0.65–5.15)	.254	2.56 (0.93–7.07)	.069
Frailty and pre-frailty	**2.73 (1.45–5.15)**	**.002**	**2.90 (1.55–5.44)**	**.001**

Results in bold are statistically significant (*P*-value < .05)

Note: Sample size was 519, with 47 deaths

**Table 4:** Multivariable cox regression of the association between fear of falling at baseline and mortality during 6-year follow-up in women

	FES-I		SQ-FAR	
	Hazard ratio (95% CI)	*P* value	Hazard ratio (95% CI)	*P* value
FES-I			–	
Low concern of falling	Reference			
Moderate concern of falling	0.65 (0.28–1.50)	.315		
High concern of falling	1.21 (0.41- 3.53)	.727		
SQ-FAR	–			
No FOF			Reference	
FOF without AR			0.69 (0.30–1.62)	.398
FOF with AR			2.13 (0.86- 5.30)	.104
Depression	1.58 (0.79–3.16)	.191	1.50 (0.76–2.99)	.245
Visual impairment	1.32 (0.61–2.89)	.482	1.29 (0.59–2.82)	.518
BADL impairment	1.05 (0.41–2.68)	.920	1.06 (0.42–2.64)	.906
IADL impairment	2.29 (0.90–5.83)	.083	1.76 (0.70–4.42)	.233
Frailty and pre-frailty	1.23 (0.59–2.58)	.578	1.10 (0.53–2.31)	.799

Note: Sample size was 719, with 39 deaths

## Discussion

This study shows that the highest level of FOF, measured with the FES-I or the SQ-FAR, is associated with the 6-year mortality only in unadjusted analyses among young-old community-dwelling people.

A significant contribution of this study is thus to clarify, in a large sample of older adults, that FOF is not an independent risk factor for mortality when adjusting for potential confounders pertaining to gender, cognition, visual and functional impairment, as well as frailty. These findings further strengthen those of another study (Oh et al. 2019) but contrast with those of some other previous studies (H.-T. Chang et al. 2017; Kim & Bae 2020; Lee et al. 2020). This may be explained by several reasons. First, in this population of young-old people aged 66 to 71 years at baseline, the mortality rate at 6 years is much lower than in these studies. Second, the way FOF was measured, in this study SQ-FAR and FES-I, as compared to a single question in previous studies may influence the results. Third, contrary to previous studies, this analysis was able to adjust for frailty status and impairment in activities of daily living, which have been identified as predictors of mortality in previous studies (S.-F. Chang & Lin 2015; Kojima et al. 2018; Stineman et al. 2012; Hennessy et al. 2015).

Although our results showed no independent association between FOF and death, reporting high levels of FOF with FES-I or SQ-FAR increased the odds of disability at 3 and 6-year follow up in the same population (Belloni et al. 2020a). Thus, being a potentially reversible factor, FOF detection in clinical practice and the subsequent implementation of tailored interventions could help to decrease the risk of negative outcomes related to FOF.

In sex specific analyses, only the highest level of FOF as assessed with FES-I was associated with the death outcome in men. This finding adds to previous evidence of a stronger association among men (H.-T. Chang et al. 2017; Kim & Bae 2020). The non-significant association among women could be related to the small number of participants reporting higher levels of fear. Further larger studies could investigate more in-depth the role of gender in the association between FOF and mortality.

A strength of this study is its prospective design in a large representative sample of young-old community-dwelling individuals. Additionally, it focuses on two simultaneous measures of FOF, that both provide information on FOF severity. Finally, data from the Lc65 + cohort allowed to adjust for several specific confounders, such as frailty status that were not accounted for in previous studies. The main limitation of this study is the relatively small number of participants who reported highest levels of FOF which, together with the low death rate at follow-up, limited the statistical precision of the estimate of associations, in particular after stratification for gender. Given the self-reported nature of the variables, some overlap between FOF measured by the FES-I and IADL, respectively BADL, cannot be excluded. Indeed, some of the 16 FES-I items are very close to those included in the measures of IADL or BADL, and some participants might not be able to differentiate the difficulties to perform such activities from the fear of falling while performing them. Nevertheless, there was no significant collinearity between the FES-I variable and the IADL, respectively BADL variable, allowing to keep the latter in the multivariable models.

As a conclusion, the results of this study in young-old community-dwelling people indicate that FOF should be considered a marker of mortality risk rather than a causal risk factor for death. Indeed, the association between a high-level of FOF, or FOF with activity restriction, and mortality disappears when adjusting for confounders.

FOF yet remains independently related to other negative health outcomes so that an early identification of older people experiencing FOF, followed by a personalized care, may still help to decrease FOF and its negative consequences.

## Data Availability

Data are not publicly available and the authors do not have permission to share data. Any request can be addressed to the corresponding author.

## Appendix

See Tables 3 and 4

## Abbreviations

FOF: Fear of falling
FES-I: Falls efficacy scale-international
SQ-FAR: Single question on fear of falling and activity restriction
Lc65 +: Lausanne cohort 65 +
BADL: Basic activities of daily living
IADL: Instrumental activities of daily living
MMSE: Folstein’s mini mental state examination

## Code availability

All statistical analyses were conducted using Stata, version 14.0.
